# Malignant Mesothelioma of the Tunica Vaginalis: Presenting with Intermittent Scrotal Pain and Hydrocele

**DOI:** 10.1155/2012/189170

**Published:** 2012-07-18

**Authors:** Tarık Esen, Omer Acar, Kamil Peker, Kemal Sarman, Ahmet Musaoglu, Ahmet Tefekli

**Affiliations:** ^1^School of Medicine, Koc University, 34450 Istanbul, Turkey; ^2^Division of Urology, VKF American Hospital, 34365 Istanbul, Turkey; ^3^Istanbul Pathology Group, 34365 Istanbul, Turkey

## Abstract

Paratesticular mesotheliomas are very rare tumors. In this paper, we present the management of a 38-year-old male patient with paratesticular malignant mesothelioma who was initially misdiagnosed and treated as recurrent epididymitis. After the final pathology report defining paratesticular mesothelioma during scrotal exploration, he underwent radical orchiectomy and hemiscrotal excision as a complementary, secondary procedure. His metastatic workup did not show any dissemination. Therefore, he did not receive any adjuvant treatment and remained disease-free for more than 2 years.

## 1. Introduction

Only a subset of the tumors within the scrotal sac is extratesticular. The paratesticular region, including the spermatic cord, testicular tunics, epididymis and vestigial remnants, is composed of a mixture of epithelial, mesothelial, and mesenchymal elements [1]. With the exception of cystadenomas of the epididymis, occasional dermoid cysts of the spermatic cord and rare papillary tumours, most tumours involving the testicular adnexia are of mesenchymal origin and harbor malignant potential [2, 3].

Herein, we give the clinical details of a patient with paratesticular malignant mesothelioma, who presented with a longstanding history of scrotal pain and swelling and received many medications due to the presumptive misdiagnosis of epididymitis before the final diagnosis of mesothelioma.

## 2. Case Report

 A 38-year-old male patient presented with left testicular pain and swelling. His past medical history was unremarkable. However, he had similar complaints which also included intermittent swelling for almost 1.5 years, and during this period he received many medications for recurrent epididymitis. 

Previous imaging findings and serum tumor marker values obtained elsewhere were within normal limits. Physical examination was completely normal on his initial visit in our department, upon which he was recommended to present while he has the scrotal swelling again. The patient represented 1 month later with a significant, soft, noninfectious swelling on the left hemiscrotum, suggesting reactive hydrocele formation. Left epididymis was minimally dilated and sensitive upon palpation.

 Scrotal ultrasonography demonstrated findings related to hydrocele and left epididymitis. Testicular parenchyma was devoid of any ultrasonographic abnormality. Blood and urine tests, including serum tumor markers, were again within normal limits.

**Figure 1:**
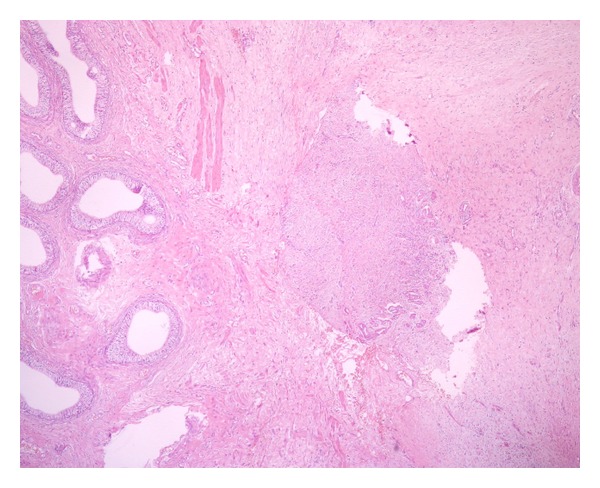
Low-power view of infiltrating malignant mesothelial tubular structures abutting the scrotal wall at the level of tunica dartos in close proximity of the epididymis (×40 magnification; H&E staining).

**Figure 2:**
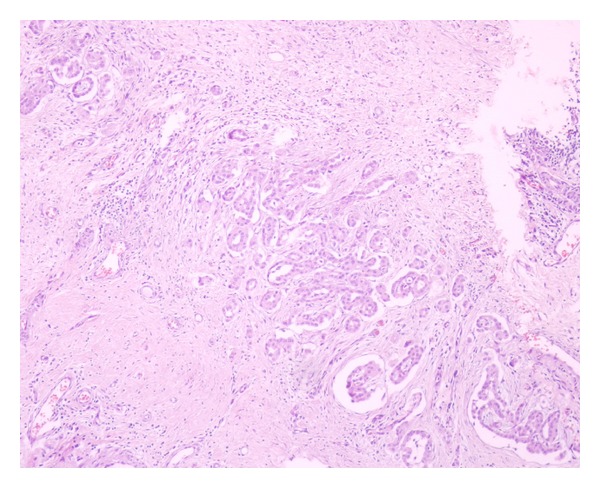
Intermediate-power view of complex, interconnecting pseudoglandular mesothelial proliferation spraying out into abortive tubular structures and rare isolated cells in a desmoplastic stroma. Neoplastic mesothelial cells are characterised by higher nucleus/cytoplasm ratio than normal cells and have prominent nucleoli and eosinophilic cytoplasm disposed into strands with nuclear overlapping (×100 magnification; H&E staining).

**Figure 3:**
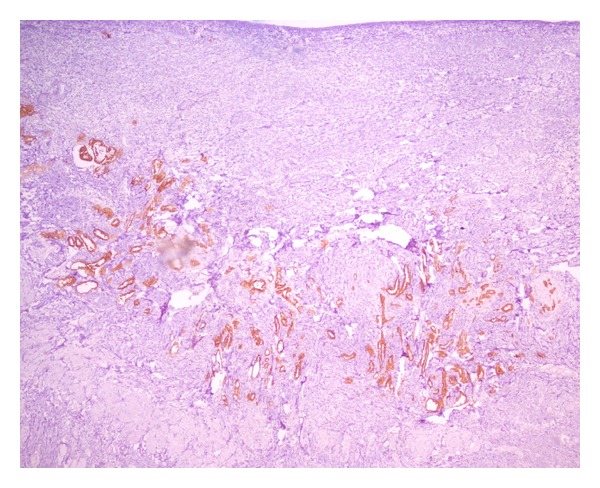
Calretinin immunohistochemistry highlighting the muscle infiltrative properties of the neoplastic mesothelial cells in deep submesothelial lamina propria (×400 magnification; H&E staining).

 Based on these findings, he was scheduled for left hemiscrotal exploration, during which, inflammatory tunical layers together with caput epididymis, which seemed dilated and inflamed, were excised and sent to pathology. Postoperative course was uneventful. He was discharged the following day. Histopathological examination of the excised specimen revealed normal findings for epididymis but epitheloid-type paratesticular mesothelioma (Figure 1) originating from tunica vaginalis to everybody's surprise. Neoplastic mesothelial cells were characterized by higher nucleus/cytoplasm ratio than normal cells, prominent nucleoli and eosinophilic cytoplasm (Figure 2). Diffuse expression of cytokeratin 5/6 and calretinin (Figure 3) by the tumor cells as well as the muscle infiltrative properties of the neoplastic mesothelial cells in deep submesothelial lamina propria confirmed our diagnosis of malignant mesothelioma, which measured 3 mm on its maximum diameter.

 After consulting the case with medical oncology and radiation oncology departments, we decided to perform left radical orchiectomy and hemiscrotectomy as a complementary procedure. Chest and abdominal computed tomographies (CT) as well as positron-emission tomography (PET) scans were all negative. After the second operation, he was hospitalized for 6 days due to the development of left hemiscrotal hematoma, which was managed conservatively. Pathological examination of the specimens removed during the second operation confirmed the diagnosis of malignant paratesticular mesothelioma originating from the tunica vaginalis. 

Although he was offered active surveillance, he asked for a second consultation in an urooncology center elsewhere, at which he was recommended to have a diagnostic laparoscopy to exclude any lymphatic spread after having confirmed the histopathologic diagnosis of paratesticular malignant mesothelioma by second opinion. He had the procedure where all biopsies taken from pelvic lymph nodes were found to be negative. He was then given no additional treatment. 

The patient is still under our close followup, and he is disease-free for 26 months. 

## 3. Discussion

Mesotheliomas are relatively rare tumors that arise from the serosal surface of the pleura, peritoneum, and pericardium. On rare occasions, they originate from the tunica vaginalis of the testis in which case they manifest as a paratesticular mass [4]. Since the first description published by Barbera and Rubino in 1957 [5], a total of 234 cases have been described so far [4, 6]. Paratesticular mesotheliomas are rare tumors, accounting for around 0.3% to 1.4% of all cases of malignant mesotheliomas [6]. Being morphologically identical to mesotheliomas at other body parts, paratesticular mesotheliomas are known to be aggressive tumors [7–11]. They may present at any age but the peak incidence is in the sixth to eighth decades of life. Most tumours originate from the tunica vaginalis. Asbestos exposure is the main risk factor for developing malignant mesothelioma. However, a correlation between asbestos exposure and mesothelial neoplastic proliferation could be documented in only less than half of the cases [12]. A number of other agents have been implicated in the development of malignant mesothelioma such as erionite and fluoroedenite or ionising radiation with the use of thorotrast [13]. Moreover, genetic factors may play a contributive role, consistently with familial clusters in certain regions of Turkey [14]. Our patient denied any environmental, occupational, or domestic exposure to the documented etiologic factors. Moreover, his family history was unremarkable. 

Apart from a past medical history consistent with exposure to asbestos or other natural fibers, preoperative clinical findings usually could not point out the diagnosis of malignant mesothelioma. Firm, painless scrotal mass accompanied by a reactive hydrocele is the usual presentation. The hydrocele or the mass itself may gradually increase in size [3]. The diagnosis can sometimes be made only after presentation with a recurrent hydrocele or invasion of the scrotum [15]. Similarly, our case had a history of longstanding scrotal pain and swelling suggestive of epididymitis. 

Suspected diagnosis should be confirmed with imaging modalities such as ultrasonography and computerized tomography. On ultrasonography, malignant mesothelioma is usually characterized by a hypoechoic hydrocele with heterogeneous masses of increased echogenicity at the periphery [16]. However, ultrasonographic appearance may not be conclusive about the nature of the disease process. This was the fact in our patient whose ultrasonographic findings were limited to moderately enlarged hydrocele and epididymal engorgement.

Radical inguinal orchiectomy is accepted as the optimal treatment for paratesticular malignant mesothelioma [12]. However, given the inconclusive ultrasonographic appearance and chronic hemiscrotal symptomatology, we decided to proceed with surgical exploration. Epididymal inflammatory changes, which were most prominent in the caput region, were noted, and caput epididymis was excised in order to document the underlying pathology. Histopathological findings were consistent with malignant mesothelioma originating from the tunica vaginalis. Although, tumor seemed to be localized to paratesticular tissues both clinically and radiographically, radical excision (orchiectomy and hemiscrotectomy) was performed as an additional procedure due to the possible aggressive clinical behaviour.

 Although the followup in published series is often short and variable, almost half of the patients have been reported to develop recurrence or metastasis after radical orchiectomy for paratesticular malignant mesothelioma. Local recurrence after orchiectomy is reported in approximately 10% of the patients [12]. Moreover approximately 40% of the patients die from their disease, with a median survival of 24 months [12, 17]. Given these relatively poor outcomes, some authors proposed adjuvant radiotherapy or retroperitoneal lymph node dissection [18]. Similarly, diagnostic laparoscopy, which was performed for staging purposes in another center after confirming the diagnosis, should have been offered with these facts in mind. However, strict recommendations about the role of adjuvant therapy or staging laparoscopy in the management of paratesticular malignant mesothelioma cannot be made based upon these limited data. We opted for active surveillance for this particular patient who accepted to comply with the follow-up protocol. He is disease-free and alive at 26 months of followup without any adjuvant treatment, which is above the median survival duration reported in the literature.

In conclusion, paratesticular neoplasms should always be kept in mind while evaluating chronic, recurrent hemiscrotal symptoms. These tumors may not be distinguishable from testicular counterparts based on preoperative findings. However once suspected, interventional measures should be prompted since delays in diagnosing paratesticular tumors may have prognostic implications.
